# A case of idiopathic giant megacolon in an obese patientitle of image

**DOI:** 10.11604/pamj.2018.31.206.16982

**Published:** 2018-11-26

**Authors:** Pietro Fransvea, Francesco Cortese

**Affiliations:** 1 Faculty of Medicine and Psychology, "La Sapienza" University of Rome-St Andrea’s Hospital, Rome, Italy; 2 Emergency Surgery and Trauma Care Unit, St Filippo Neri Hospital, Rome, Italy

**Keywords:** Megacolon idipathic obese, obese patientitle, Rome

## Image in medicine

43-year-old man complaining of abdominal nausea starting 15 days earlier and vomiting was admitted to our emergency department. The patient was affected by a sever obesity with a BMI of 55.5; there was also an history of diabetes and hypertension. The abdomen was distended, diffusely painful, tympanic to percussion and the Blumberg sign was intensely positive; WBC count was 24,000x10^3^ with marked neutrophilia, hemoglobin 9.6 g/dl, with haematocrit 30.6%. A distended colon was present at plain RX abdomen while the CT cannot be performed due to the high BMI of the patient. At laparotomy a giant idiopathic megacolon was found and an Hartmann procedure was performed. The patient was discharged 15 postop with no complication. The anatomopathological examination documented a normal colon tissue. Diagnosis in obese patients is often difficult because they are paucisintomatic and the physical examination is difficult to achieve.

**Figure 1:**
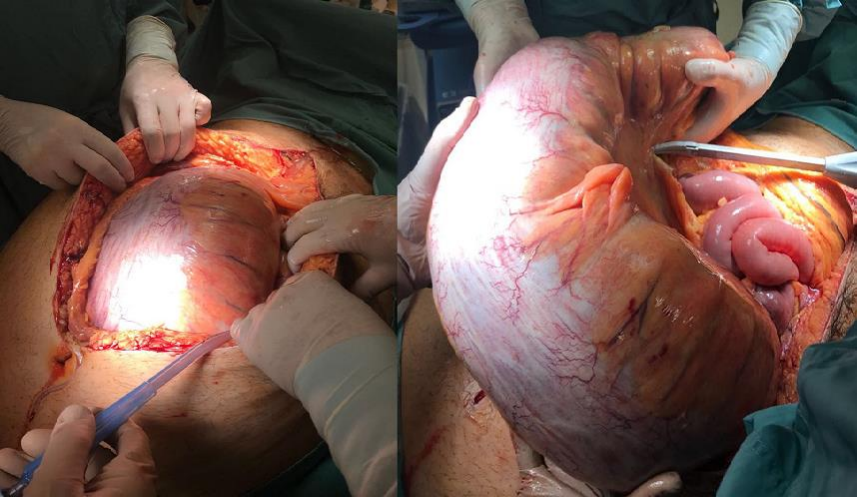
Findings at laparotomy, giant megacolon

